# An Algorithm to Predict Success of Indirect Decompression Using the Extreme Lateral Lumbar Interbody Fusion Procedure

**DOI:** 10.7759/cureus.317

**Published:** 2015-09-08

**Authors:** Brandon C Gabel, Reid Hoshide, William Taylor

**Affiliations:** 1 Neurosurgery, UC San Diego

**Keywords:** xlif, lateral, lumbar, fusion, indirect decompression, llif, laminectomy, facet fusion

## Abstract

Purpose: The purpose of this study is to define an algorithm that will predict the success of indirect decompression without the need for direct decompression in patients undergoing lateral lumbar interbody fusions.

Methods and Materials: A prospective cohort study was undertaken for patients undergoing indirect decompression with lateral lumbar interbody fusion. Patients had to meet the following criteria prior to indirect fusion: lack of facet fusion on CT, absence of free disc fragment or compressive facet joint cyst on MRI, absence of frank osteoporosis, lack of congenital and/or severe spinal stenosis on MRI, and significant reduction (greater than 50%) in leg and back pain at rest. We then assessed which patients at follow-up required a second stage open decompression procedure because of continued back and/or leg pain.

Results: Our series included 28 patients who underwent indirect decompression with extreme lateral lumbar interbody fusion. Of the 28 patients, one patient required a second stage open decompression at follow-up. The most common level operated on was the L4-L5 level. Twelve patients underwent more than a single level fusion. Average preoperative lumbar lordosis was 29 degrees and average postoperative lordosis was 45 degrees. The average patient age was 66.3 years and average follow-up was 1.21 years.

Conclusions: Our algorithm can be used as an aid to assess which patients may benefit from indirect decompression alone, compared to indirect decompression combined with posterior decompression procedures.

## Introduction

Central canal stenosis, lateral recess stenosis, and foraminal stenosis occur as a result of degenerative disc disease. Degeneration of the intervertebral discs in combination with disc herniation and concomitant osteophyte formation of the adjacent vertebral bodies is a well-known cause of stenosis in the lumbar spine. Spinal stenosis of any type is a well-known cause of disability throughout the world.  

Open decompressive procedures, including laminectomy and foraminotomy, have been widely used with success for the treatment of all types of lumbar stenosis [1]. However, traditional open surgical procedures require significant muscle dissection and disruption of the posterior ligaments. Sometimes aggressive removal of the medial facet is necessary to adequately decompress the nerve in foraminal stenosis; as a result, iatrogenic instability may occur necessitating second stage fusion procedures. In addition, decompression procedures by themselves do not restore disc height.

An alternative to open decompression is indirect decompression. With the advent of minimally-invasive lateral lumbar interbody devices, the height of the disc space can now be restored. Cage placement increases foraminal height without the need for direct decompression. Several authors have looked at the use of indirect decompression as an alternative to open laminectomy or foraminotomy. Many of these surgeons have found significant benefit to indirect decompression [2-5]. Numerous radiographic studies have shown significant improvement in foraminal height, posterior disc height, and thecal sac area with indirect decompression [2, 4-8].

Despite numerous papers discussing the benefits of indirect decompression, there has yet to be an algorithmic approach to the use of this technology. In this paper, we developed an algorithm that can be used to select patients that we feel will best benefit from an initial trial of indirect decompression. We discuss two cases, one an ideal case for indirect decompression, and the other, a poor choice for indirect decompression based on our algorithm. We also discuss our series of patients who have undergone indirect decompression alone and discuss the cases in our series that have required revision open decompression surgery for residual leg pain. 

## Materials and methods

A prospective cohort study was undertaken for patients undergoing indirect decompression with a lateral lumbar interbody procedure. Patients had to meet the following criteria prior to indirect fusion: (1) a lack of facet fusion on CT, (2) the absence of a free disc fragment or compressive facet joint cyst on MRI, (3) the absence of frank osteoporosis (Z score -2.5 or less), (4) the lack of congenital and/or severe spinal stenosis on MRI (defined as complete lack of CSF signal on T2-weighted MRI), and (5) a significant reduction (greater than 50%) in leg and back pain at rest. We then assessed which patients at follow-up required a second stage open decompression procedure because of continued back and/or leg pain.  

The following demographic and clinical data were collected: age at the time of surgery, length of follow-up, primary diagnosis at the time of surgery, levels fused, pre- and postoperative lumbar lordosis, and the condition at follow-up were recorded.

The University of California at San Diego Internal Review Board approved this retrospective study (approval #141173X). Signed informed patient consent was obtained from all patients at the time of their treatment.

## Results

Twenty-eight patients were identified who underwent primary lateral interbody fusion with the goal of indirect decompression. There were 20 females and eight males. The average age of the patients was 66.3 years (median 66.2 years). The average length of follow-up was 1.21 years (range of nine days to 4.22 years, median 0.72 years) (Table 1).

**Table 1:** Demographics and Levels Fused

Characteristic	Statistics (% of total)
Total number of patients	28 (100%)
Females	20 (71%)
Average / median age	66.3 / 66.3 years
Average / median length of follow up	1.21 / 0.72 years
Levels treated	
L1-L2 (alone)	0 (0%)
L2-L3 (alone)	0 (0%)
L3-L4 (alone)	3 (10.7%)
L4-L5 (alone)	13 (46.4%)
Two levels	3 (10.7%)
Three levels	4 (14.3%)
Greater than three levels	5 (17.9%)
Percutaneous pedicle screw fixation	21 (75.0%)
Lateral plate fixation	7 (25%)

There were zero patients who underwent single-level fusions at L1-L2 or L2-L3. Three patients underwent fusion at L3-L4 and 13 patients underwent fusion at L4-L5. Three patients underwent fusion at two contiguous spinal levels, four patients underwent fusion at three contiguous spinal levels, and five patients had fusions of more than three contiguous spinal levels. For patients with both pre- and postoperative imaging available, the average preoperative lumbar lordosis was 29 degrees and the average postoperative lumbar lordosis was 45 degrees (Table 1).

Only one patient required delayed decompression. This patient originally underwent an L4-L5 XLIF and had a delayed laminectomy at the same levels 1.3 years later for recurrent leg pain. One patient had an adjacent level XLIF performed four years after his index case but did not require direct decompression.

## Discussion

### Case illustrations                

**Figure 1:**
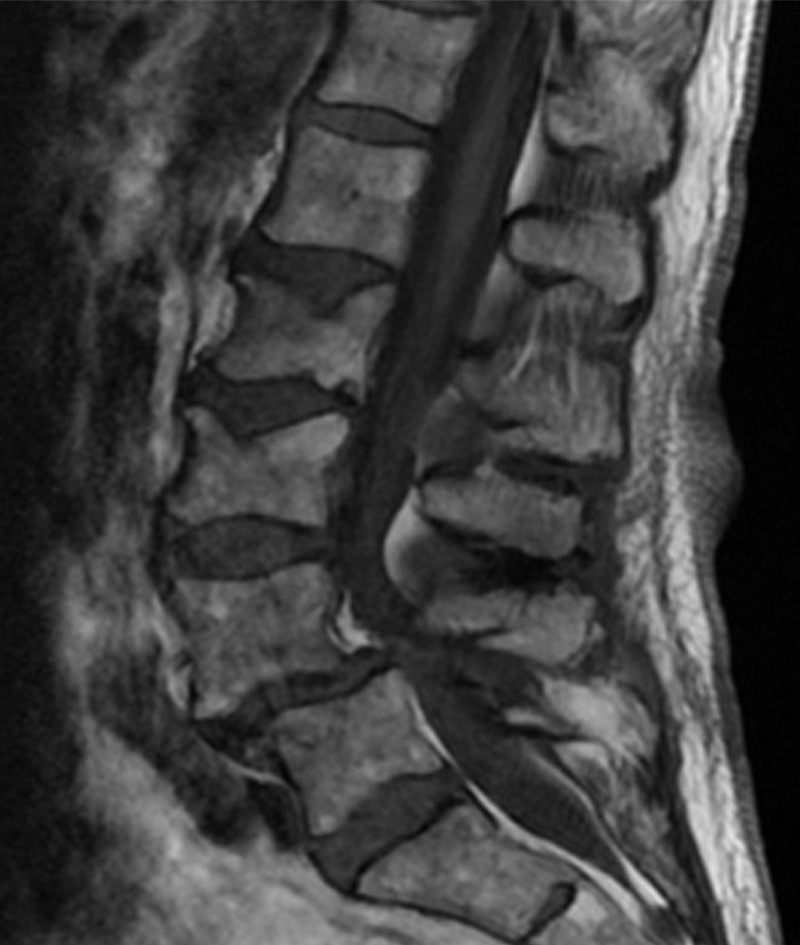
MRI of a Poor Candidate This MRI shows features of a poor candidate for indirect decompression. Note the high-grade central canal stenosis.

**Figure 2:**
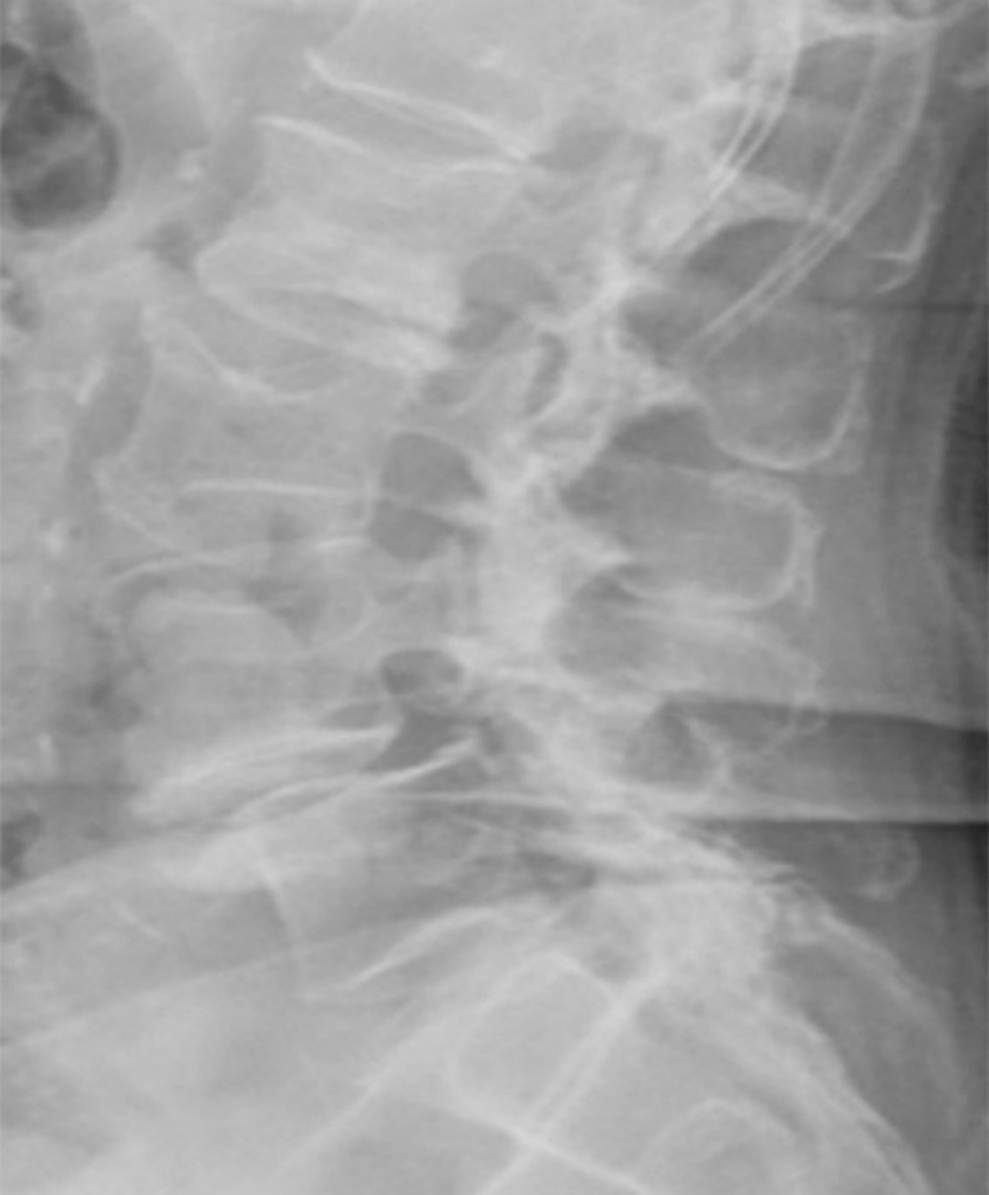
X-ray of Poor Candidate Lateral x-ray shows osteopenia/osteoporosis, which can result in interbody subsidence and need for concomitant direct decompression procedures.

Poor Patient Selection: 

The first patient is an 81-year-old female with a T-score of -2.6, severe lumbar stenosis on MRI, fused facets on CT, and bilateral radicular symptoms. MRI revealed significant stenosis secondary to L4-L5 spondylolisthesis (Figure 1). Preoperative x-rays showed poor bone quality consistent with her known osteoporosis (Figure 2). Preoperative CT scan showed evidence of fused facet joints.

This patient is unlikely to obtain relief from indirect decompression alone. Her severe central canal stenosis cannot be addressed by indirect decompression. Additionally, her poor bone quality will likely result in subsidence of lateral interbody cages with resulting collapse of her neural foramen. Furthermore, a fusion of her facet joints prevents indirect decompression of her foramen. 

**Figure 3:**
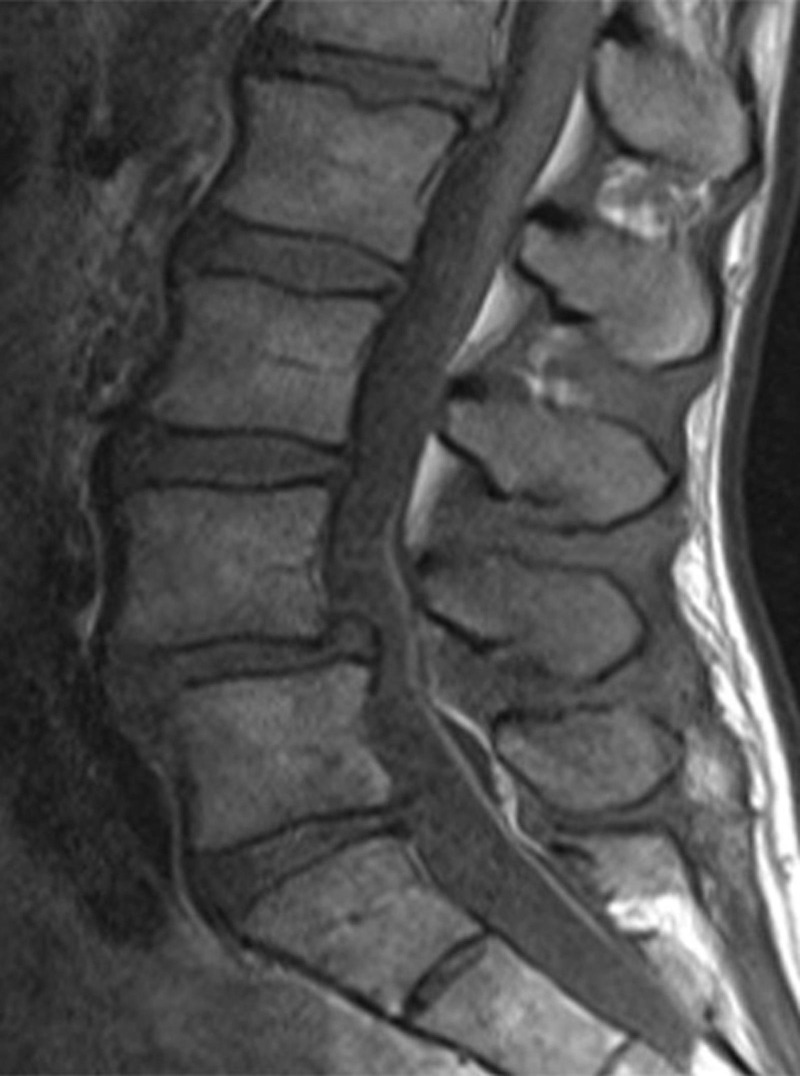
MRI of a Strong Candidate This MRI shows features of a good candidate for indirect decompression. Note the lack of central canal stenosis.

**Figure 4:**
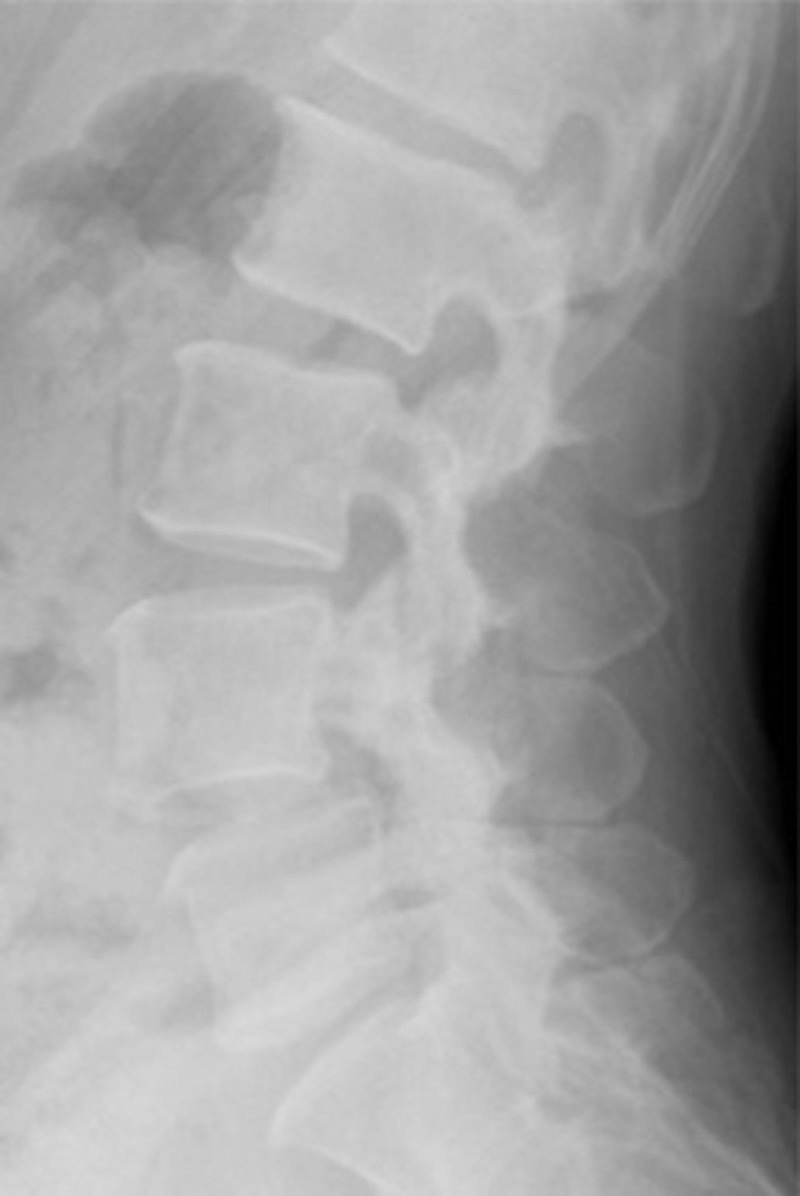
X-ray of a Strong Candidate Lateral x-ray shows good bone quality, which aids interbody fusion.

Strong Patient Selection: 

The second patient is a 43-year-old former triathlete in excellent health who presents with low back pain and bilateral radicular pain. MRI revealed stenosis at the L4-L5 level secondary to a Grade I spondylolisthesis (Figure 3). Preoperative x-rays show good bone quality (Figure 4). Preoperative CT scan did not show evidence of fused facet joints.  

This patient is likely to do well with indirect decompression alone. The good bone quality and lack of facet fusion seen on x-ray will prevent cage subsidence and prevent the collapse of the neural foramen. Her central canal stenosis is also not severe and does not need to be treated with open decompression. 

### Defining an algorithm to predict success 

Although there are many studies that have shown that indirect decompression is a viable alternative to open decompression procedures, the ideal patient for this procedure has yet to be well-defined. One study found that missed unstable spondylolisthesis, bony lateral recess stenosis, and misaligned cages were the causes of failure [5, 8]. This study concluded that bony lateral recess stenosis or unstable degenerative spondylolisthesis may benefit from direct decompression procedures in conjunction with lateral lumbar interbody fusion.

Indirect decompression relies on interbody graft placement and concomitant distraction of the neural foramen. Osteoporosis negatively affects fusion rates and may increase the risk of graft subsidence. One study found that successful posterolateral lumbar interbody fusion was more likely in patients with higher Hounsfield units on preoperative CT [9]. We do not recommend indirect decompression alone in patients with frank osteoporosis (defined as a Z-score of -2.5 or lower) because of the higher risk of failed fusion and potential for subsidence, both of which can contribute to the collapse of the neural foramen.

Pre-existing facet fusion prevents distraction of the neural foramen during cage placement. In our algorithm, patients with evidence of facet fusion on preoperative CT are considered poor candidates. Similar to the rationale for osteoporosis, facet fusion prevents distraction of the neural foramen during graft insertion. In these patients, we recommend a lateral interbody fusion with open decompression, or an open decompression alone depending on the spinal pathology being addressed.

Although the lateral approach allows the placement of relatively large cages, by itself, this approach is unable to address severe spinal stenosis or compressive lesions, such as facet cysts or extruded disc fragments. Patients with severe stenosis (defined as a complete loss of cerebrospinal fluid signal on preoperative MRI) or facet cysts causing nerve root compression were managed with direct decompression procedures with or without the addition of lateral fusion.

Interestingly, none of the patients who underwent multilevel indirect decompression required delayed direct decompression. It would seem logical that patients harboring multi-level adult degenerative disease would be at higher risk for needing concomitant direct decompression; however, in our series, none of the 12 patients who had two levels or more fused required delayed decompression at follow-up. The reason for this finding is unclear and warrants further study.

One patient required delayed open decompression at the same level. The patient had originally undergone an L4-L5 lateral fusion with percutaneous pedicle screw fixation. She had significant improvement in her back and leg pain postoperatively. After roughly six months, the patient started to develop recurrent leg pain. This was initially managed conservatively without improvement. She elected to undergo open decompression 1.3 years after the initial fusion. She did well with open decompression reporting improvement in leg pain at follow-up.

One patient who underwent a three-level lateral fusion with percutaneous screw placement developed adjacent level disease above the level of the fusion. He did well after his initial fusion with improvement in back and leg pain. Four years later, the patient presented with band-like symptoms in the lower abdomen and inguinal crease. He was found to have adjacent level disease at T12-L1 and L1-L2. He underwent indirect decompression at these levels and did well at follow-up with an improvement of his symptoms. Therefore, repeat indirect decompression at adjacent levels may be suitable in select patients.  

Our study is limited by the fact that it is a retrospective chart review on a relatively small number of patients. Despite the weaknesses of the study, only one patient required delayed open decompression surgery at follow-up. As such, we feel that using the above factors provides a relatively simple clinical and radiographic algorithm for which to select patients for indirect versus direct decompression. However, higher quality studies adjusting for other demographic, medical, and radiologic parameters will better delineate the best patient population for indirect minimally invasive decompression. 

## Conclusions

Lack of facet fusion, osteoporosis, severe lumbar stenosis, free disc fragment/facet cyst, and an improvement in low back and/or leg pain of greater than 50% during rest may be used as a guide to help determine which patients will benefit most from indirect decompression using an extreme lateral lumbar interbody approach. 
